# Prospective signs of cleidocranial dysplasia in Cebpb deficiency

**DOI:** 10.1186/1423-0127-21-44

**Published:** 2014-05-13

**Authors:** Boyen Huang, Katsu Takahashi, Ernest A Jennings, Pongthorn Pumtang-on, Honoka Kiso, Yumiko Togo, Kazuyuki Saito, Manabu Sugai, Shizuo Akira, Akira Shimizu, Kazuhisa Bessho

**Affiliations:** 1 School of Medicine and Dentistry, James Cook University, Cairns, Australia; 2 Department of Oral and Maxillofacial Surgery, Graduate School of Medicine, Kyoto University, Shogoin-Kawahara-cho 54, Sakyo-ku, Kyoto 606-8507, Japan; 3 Setthasiri Animal Hospital, Bangkok, Thailand; 4 Translational Research Center, Kyoto University Hospital, Kyoto University, Kyoto, Japan; 5 Laboratory of Host Defense, World Premier International Immunology, Frontier Research Center, Osaka University, Osaka, Japan

**Keywords:** Cebpb, Cleidocranial dysplasia, Clavicle, Thoracic cage, Zygomatic arch, Masseter, Temporal muscle

## Abstract

**Background:**

Although runt-related transcription factor 2 (RUNX2) has been considered a determinant of cleidocranial dysplasia (CCD), some CCD patients were free of RUNX2 mutations. CCAAT/enhancer-binding protein beta (Cebpb) is a key factor of Runx2 expression and our previous study has reported two CCD signs including hyperdontia and elongated coronoid process of the mandible in Cebpb deficient mice. Following that, this work aimed to conduct a case-control study of thoracic, zygomatic and masticatory muscular morphology to propose an association between musculoskeletal phenotypes and deficiency of Cebpb, using a sample of Cebpb^-/-^, Cebpb^+/-^ and Cebpb^+/+^ adult mice. Somatic skeletons and skulls of mice were inspected with soft x-rays and micro-computed tomography (μCT), respectively. Zygomatic inclination was assessed using methods of coordinate geometry and trigonometric function on anatomic landmarks identified with μCT. Masseter and temporal muscles were collected and weighed. Expression of Cebpb was examined with a reverse transcriptase polymerase chain reaction (RT-PCR) technique.

**Results:**

Cebpb^-/-^ mice displayed hypoplastic clavicles, a narrow thoracic cage, and a downward tilted zygomatic arch (p < 0.001). Although Cebpb^+/-^ mice did not show the phenotypes above (p = 0.357), a larger mass percentage of temporal muscles over masseter muscles was seen in Cebpb^+/-^ littermates (p = 0.012). The mRNA expression of Cebpb was detected in the clavicle, the zygoma, the temporal muscle and the masseter muscle, respectively.

**Conclusions:**

Prospective signs of CCD were identified in mice with Cebpb deficiency. These could provide an additional aetiological factor of CCD. Succeeding investigation into interactions among Cebpb, Runx2 and musculoskeletal development is indicated.

## Background

Cleidocranial dysplasia (CCD) is a congenital skeletal disease typically manifesting cranial deformity, clavicle hypoplasia and unerupted supernumerary teeth [1-5]. Some human CCD cases also displayed hypotrophy of the masseter muscle, abnormality of the mandible and/or deformation of the zygomatic arch [3-5]. Haploinsufficiency of the runt-related transcription factor 2 (RUNX2 in humans, Runx2 in mice) has been considered a determinant of CCD in humans [6]. A recent study has also identified bony and dental defects consistent with signs of human CCD amongst mice with impaired activity of the Runx2 P1 promoter [7]. Expression of the Runx2 P1 promoter in the developing skull has been confirmed [8] and its activity is essential to establishing a sufficient number of osteoprogenitor cells for normal skeletogenesis [7]. Although the mechanism of CCD-related dental anomalies is not clear, induction of the dentition was associated with fibroblast growth factor (FGF) signaling [9] that was mediated by Runx2 during development of teeth [10] and bone [11]. Nevertheless, about one-third of CCD patients were free of RUNX2 mutations [6]. Furthermore, diverse dental manifestations resulting from identical mutations of the RUNX2 gene have been observed [1]. These infer potential involvement of other factors on occurrence of CCD.

CCAAT/enhancer binding protein beta (CEBPB in humans, Cebpb in mice) is a transcription factor which binds to consensus sequences and affects the transcription of various genes involved in proliferation and differentiation, and found in the liver [12], the mammary gland [13] and the immune system [14,15]. In addition to its relevance in tumorigenesis [16] and adipogenesis [17], previous studies have suggested that Cebpb is a key factor of Runx2 expression in bone formation [18-21]. A reduction in Runx2 expression was accompanied by a decrease in osteogenic potential and shown to be related to ectopic expression of Cebpb [18]. On the other hand, a functional synergism of Runx2 and Cebpb during osteoblast [21] and chondrocyte differentiation [20] has been reported. Suppressed differentiation of osteoblasts and delayed chondrocyte hypertrophy due to a complete deletion of Cebpb is likely to postpone bone formation [19]. This concurs with phenotypes of p20Cebpb (a dominant negative inhibitor of Cebpb) overexpressing mice, including a reduced amount of alveolar bone and a lower bone volume fraction of the mandible [22].

Recent studies have identified multiple supernumerary teeth [23], hypoplastic clavicles [20], an open fontanelle [20] and an elongated coronoid process [23] in mice with Cebpb deficiency. These phenotypes coincide with signs of CCD in humans [1-5] and consequently a relationship between CEBPB and CCD is suspected [20]. To explore prospective signs of human CCD in a murine model, this study aimed to conduct a case-control study of thoracic, craniofacial and myological variations, using a sample of Cebpb^-/-^, Cebpb^+/-^ and Cebpb^+/+^ mice. A special interest was to investigate morphology of the zygomatic arch and mass of both masseter and temporal muscles.

## Methods

An appropriate ethics approval has been obtained from the Institutional Review Board of Kyoto University (Reference Number: Med Kyo 11518). Cebpb^+/-^ and Cebpb^-/-^ mice were generated as described previously [15]. Thirty-five adult mice were euthanised with carbon dioxide gas for inspection, including 5 Cebpb^-/-^ (5 female), 16 Cebpb^+/-^ (10 female and 6 male) and 14 Cebpb^+/+^ (8 female and 6 male) mice. All female mice were sacrificed at the age of 12 months, whilst the age of male mice used ranged from 4.5 to 13 months. Owing to a high neonatal mortality of Cebpb^-/-^ mice [24], male animals of this genotype were not attainable in this sample.

Firstly, skeletons of the female mice were imaged with soft x-rays (SOFRON; SRO-M50, Sofron X-ray Industry Corporation Ltd., Tokyo, Japan). Both lateral and dorsal-ventral radiographs of the experimental animals were evaluated by a single-blinded examiner who is a qualified veterinarian.

**Figure 1:**
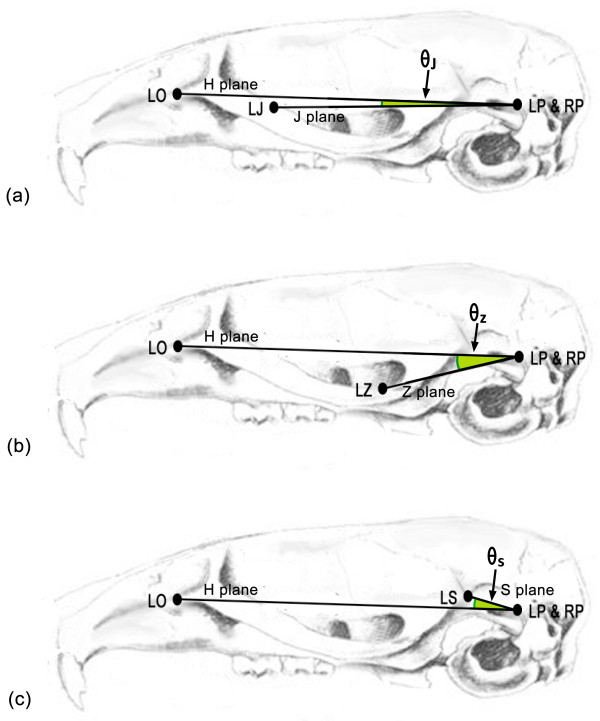
**Craniofacial landmarks, established planes and dihedral angles used in this study. (a-c)** Landmark LO, LP and RP formed a horizontal reference plane (H plane). **(a)** Landmark LJ, LP and RP formed a jugal plane (J plane). The dihedral angle between J plane and H plane was defined as θ_J_. **(b)** Landmark LZ, LP and RP formed a zygomatic plane (Z plane). The dihedral angle between Z plane and H plane was defined as θ_Z_. **(c)** Landmark LS, LP and RP formed a squamosal plane (S plane). The dihedral angle between S plane and H plane was defined as θ_S_.

Secondly, murine skulls of the 23 female mice were assessed with a micro-computed tomography (μCT) scanner (SMX-100CT-SV3; Shimadzu, Kyoto, Japan). This technique was applied to identify predetermined craniofacial landmarks including Landmark LO, LP, RP, LJ, LZ, and LS (Table 1) [23,25,26]. As the external auditory meatus of mice is located at a lower level than that of humans [27], Landmark LP and RP situated slightly superior to the external auditory meatus were used to replace human Porions (the most superior point of external auditory meatus visible on a lateral cephalometric radiograph) to establish a horizontal plane (H plane) with Landmark LO in this study. Following this, a jugal plane (J plane: defined by Landmark LJ, LP, and RP), a zygomatic plane (Z plane: defined by Landmark LZ, LP and RP), and a squamosal plane (S plane: defined by Landmark LS, LP and RP) were established (Figure 1). Usage of the dihedral angles in the assessment of human zygomatic anatomy has been reported [26]. The dihedral angles between H plane and J plane, between H plane and Z plane, and between H plane and S plane were calculated using a method of coordinate geometry and trigonometric function [28]. The following formulae were used.

**Table 1:** Murine anatomical landmarks used in this study

**Landmarks**	**Anatomical positions**
LO	Anterior notch on frontal process lateral to infraorbital fissure, left side
LP	Intersection of parietal, temporal and occipital bones, left side
RP	Intersection of parietal, temporal and occipital bones, right side
LJ	Intersection of zygomatic process of maxilla with zygoma, superior surface, left side
LZ	Intersection of zygoma with zygomatic process of temporal, superior aspect, left side
LS	Joining of squamosal body to zygomatic process of squamosal, left side

Given the equation of H plane containing Landmark LO (*x*_*LO*_, *y*_*LO*_, *z*_*LO*_), LP (*x*_*LP*_ , *y*_*LP*_ , *z*_*LP*_) and RP (*x*_*RP*_ , *y*_*RP*_ , *z*_*RP*_) was *a*1 *x* + *b*1 *y* + *c*1 *z* + *d*1 = 0, the equation of J plane covering Landmark LJ (*x*_*LJ*_, *y*_*LJ*_, *z*_*LJ*_), LP (*x*_*LP*_ , *y*_*LP*_ , *z*_*LP*_) and RP (*x*_*RP*_ , *y*_*RP*_ , *z*_*RP*_) was *a*2 *x* + *b*2 *y* + *c*2 *z* + *d*2 = 0, and the dihedral angle between H plane and J plane was θ_J_.

Where,

$$\begin{array}{l} a1=\left(y_{\mathrm{LP}}-y_{\mathrm{LO}}\right)\;(z_{\mathrm{RP}}-z_{\mathrm{LO}})-(y_{\mathrm{RP}}-y_{\mathrm{LO}})\;(z_{\mathrm{LP}}-z_{\mathrm{LO}}),b1=(z_{\mathrm{LP}}-z_{\mathrm{LO}})\;(x_{\mathrm{RP}}-x_{\mathrm{LO}})-(z_{\mathrm{RP}}-z_{\mathrm{LO}})\;(x_{\mathrm{LP}}-x_{\mathrm{LO}}),\\ c1=\left(x_{\mathrm{LP}}-x_{\mathrm{LO}}\right)\;(y_{\mathrm{RP}}-y_{\mathrm{LO}})-(x_{\mathrm{RP}}-x_{\mathrm{LO}})\;(y_{\mathrm{LP}}-y_{\mathrm{LO}}),d1=-(a1\;x_{\mathrm{LO}}+b1\;y_{\mathrm{LO}}+c1\;z_{\mathrm{LO}}),\\ a2=\left(y_{\mathrm{LP}}-y_{\mathrm{LJ}}\right)\;(z_{\mathrm{RP}}-z_{\mathrm{LJ}})-(y_{\mathrm{RP}}-y_{\mathrm{LJ}})\;(z_{\mathrm{LP}}-z_{\mathrm{LJ}}),b2=(z_{\mathrm{LP}}-z_{\mathrm{LJ}})\;(x_{\mathrm{RP}}-x_{\mathrm{LJ}})-(z_{\mathrm{RP}}-z_{\mathrm{LJ}})\;(x_{\mathrm{LP}}-x_{\mathrm{LJ}}),\\ c2=\left(x_{\mathrm{LP}}-x_{\mathrm{LJ}}\right)\;(y_{\mathrm{RP}}-y_{\mathrm{LJ}})-(x_{\mathrm{RP}}-x_{\mathrm{LJ}})\;(y_{\mathrm{LP}}-y_{\mathrm{LJ}}),d2=-(a2\;x_{\mathrm{LJ}}+b2\;y_{\mathrm{LJ}}+c2\;z_{\mathrm{LJ}}). \end{array}$$

Thus,

$$\theta_{J}=\arccos\left(\frac{a1\;a2+b1\;b2+c1\;c2}{\sqrt{a1^{2}+b1^{2}+c1^{2}}\;\sqrt{a2^{2}+b2^{2}+c2^{2}}\;}\right)$$

Likewise, the degree of the dihedral angles between H plane as well as Z plane (θ_Z_) and between H plane as well as S plane (θ_S_) was determined.

Thirdly, masseter and temporal muscles were collected from 12 male littermates and weighed according to the procedures reported in an earlier study [29]. The temporal/masseter mass percentage was calculated by dividing the weight of the left temporal muscle by the weight of the left masseter muscle and then multiplying 100.

To identify the expression of Cebpb, mRNA expression of the target gene (Cebpb) was assessed. Tissues used for this purpose were collected from the masseter muscle, the temporal muscle, the zygomatic bone, and the clavicle bone of a Cebpb^+/+^ mouse, respectively. Standard procedures for the preparation of the reverse transcriptase polymerase chain reaction (RT-PCR) were carried out as suggested in the literature [30,31]. Primers applied to perform the RT-PCR technique included murine Cebpb sense (5′-ACACGTGTAACTGTCAGCCG-3′) and murine Cebpb antisense (5′-GCTCGAAACGGAAAAGGTTC-3′). Specific primers for murine glyceraldehyde-3-phosphate dehydrogenase (Gapdh) were used to check the internal control [32]. The RT-PCR technique was conducted with a GeneAmp PCR System 9700 thermal cycler (Applied Biosystems, Foster City, CA, USA). All procedures were carried out according to the manufacturers’ instructions.

Data entry and statistical analysis were conducted with the IBM SPSS Statistics (version 20.0, IBM Corporation, Somers, NY, USA). An independent samples *t*-test method was used to assess the difference in the degree of anatomic dihedral angles between genotypes of Cebpb deficiency and the control (Cebpb^+/+^ mice) [33]. In addition, a paired samples *t*-test method was applied to examine the difference in the temporal/masseter mass percentage between Cebpb^+/-^ and Cebpb^+/+^ littermates [33]. This paired method has been used to compare dental phenotypes between twins [34]. The level of two-sided significance for all statistical procedures was set at 5%.

## Results

**Figure 2:**
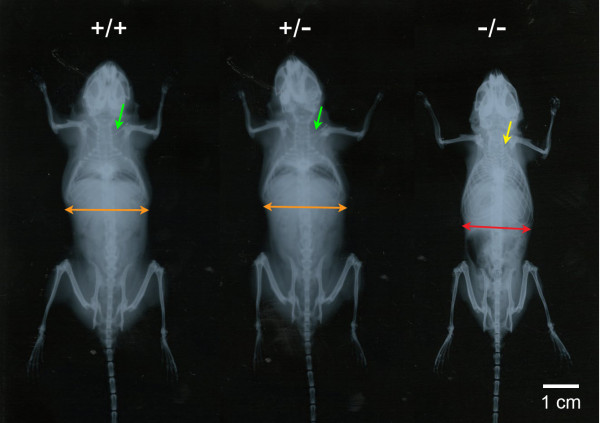
**Difference in morphology of clavicles and the thoracic cage.** The radiographic image showed a dorsal-ventral view of a Cebpb^+/+^, a Cebpb^+/-^ and a Cebpb^-/-^ 12-month-old mice in this sample. Shorter thinner clavicles with a lower radiopacity (yellow arrow) and a narrow thoracic cage (red arrow) were exclusively seen in Cebpb^-/-^ mice. Due to the limited size of a radiograph film, the radiographic images of the three mice were taken under the same condition separately. The images were displayed without transformation in dimensions, contrast, brightness and/or colour. Scale bar: 1 cm. (n = 23, including 5 Cebpb^-/-^, 10 Cebpb^+/-^ and 8 Cebpb^+/+^ female mice).

**Figure 3:**
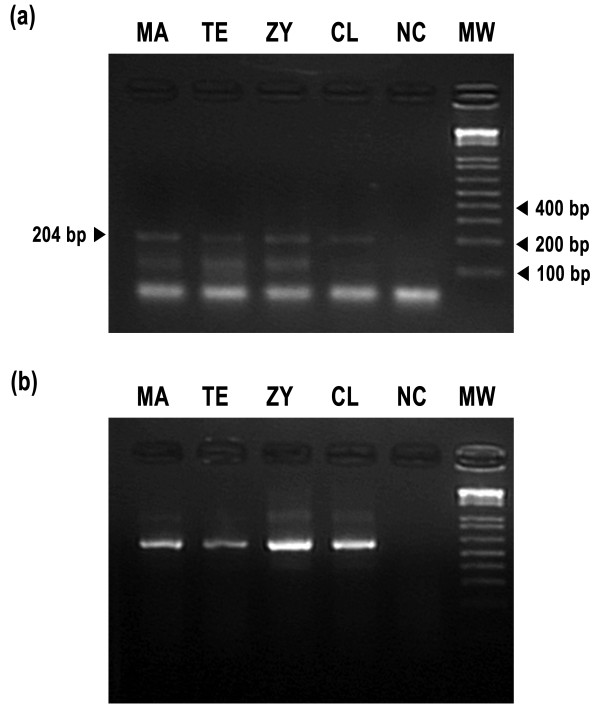
**Assessment of mRNA expression in the masseter muscle (MA), the temporal muscle (TE), the zygomatic bone (ZY), the clavicle (CL) and a negative control (NC) with a RT-PCR technique.** The size of the molecular weight markers (MW) is displayed on the right, which is given in base pairs (bp). **(a)** Cebpb expression was identified in MA, TE, ZY and CL of a Cebpb^+⁄+^ adult mouse. **(b)** Gapdh, as an internal control, was detected in the same sites of the mouse.

Shorter thinner clavicles with a lower radiopacity and a narrower thoracic cage were identified exclusively in Cebpb^-/-^ mice but not Cebpb^+/-^ and/or Cebpb^+/+^ mice (Figure 2). A normal morphology of limb joints was seen in radiographs from animals of all three genotypes. Of further note, mRNA expression of Cebpb was found in the clavicle, the zygoma, the temporal muscle and the masseter muscle (Figure 3).

**Figure 4:**
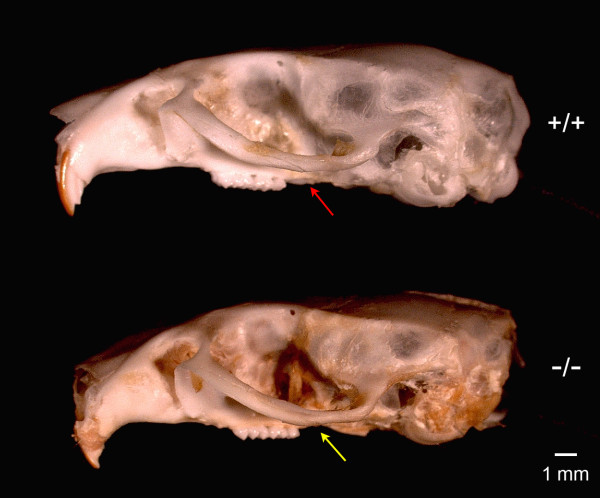
**Difference in tilt angulation of the zygomatic arch.** The photographic image showed a lateral view of dry skulls respectively collected from a Cebpb^+/+^ and a Cebpb^-/-^ 12-month-old mice. A downward tilt of the zygomatic arch in the Cebpb^-/-^ mouse was confirmed by the morphology of the dry skull (yellow arrow). Scale bar: 1 mm. (n = 23, including 5 Cebpb^-/-^, 10 Cebpb^+/-^ and 8 Cebpb^+/+^ female mice).

The mean measurement of θ_Z_ was 16.3 ± 1.1 degree, 13.0 ± 1.1 degree and 13.4 ± 1.0 degree in Cebpb^-/-^, Cebpb^+/-^ and Cebpb^+/+^ mice, respectively (Table 2). Those animals with a Cebpb^-/-^ genotype showed a larger degree of θ_Z_ than that were from a Cebpb^+/+^ (t = 4.964, df = 11, p < 0.001) or a Cebpb^+/-^ genetic background (t = 5.429, df = 13, p < 0.001) (Table 2). The latter two genotypes did not differ in the degree of θ_Z_ (p = 0.357) (Table 2). The degree of θ_J_ in Cebpb^-/-^, Cebpb^+/-^ and Cebpb^+/+^ subjects was separately 6.2 ± 1.1 degree, 5.5 ± 0.7 degree and 6.3 ± 1.7 degree (p ≥ 0.142) (Table 2). The degree of θ_S_ in Cebpb^-/-^, Cebpb^+/-^ and Cebpb^+/+^ mice was 3.7 ± 2.2 degree, 3.1 ± 2.2 degree and 5.1 ± 3.3 degree, individually (p ≥ 0.148) (Table 2). Figure 4 demonstrates a lateral view of dry skulls of a Cebpb^-/-^ and a Cebpb^+/+^ 12-month-old female mice.

**Table 2:** Means of dihedral angles by genotypes in 12-month-old female mice

		**Cebpb**^**+/+**^**(n = 8)**	**Cebpb**^**+/-**^**(n = 10)**	**Cebpb**^**-/-**^**(n = 5)**	**All (n = 23)**
θ_J_		6.3 ± 1.7	5.5 ± 0.7	6.2 ± 1.1	5.9 ± 1.2
p values	^+/+^vs ^+/-^	0.227			
	^+/-^ vs ^-/-^		0.142		
	^-/-^ vs ^+/+^			0.923	
θ_Z_		13.4 ± 1.0	13.0 ± 1.1	16.3 ± 1.1	13.9 ± 1.7
p values	^+/+^vs ^+/-^	0.357			
	^+/-^ vs ^-/-^		<0.001*		
	^-/-^ vs ^+/+^			<0.001*	
θ_S_		5.1 ± 3.3	3.1 ± 2.2	3.7 ± 2.2	3.9 ± 2.7
p values	^+/+^vs ^+/-^	0.148			
	^+/-^ vs ^-/-^		0.432		
	^-/-^ vs ^+/+^			0.633	

*p < 0.001.

**Figure 5:**
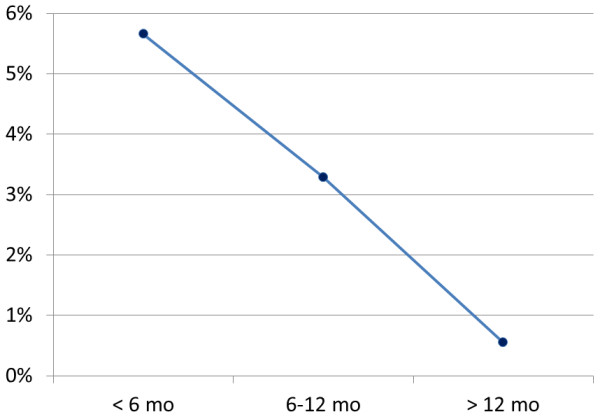
**Difference in the temporal/masseter mass percentage between Cebpb**^**+/-**^**and Cebpb**^**+/+**^**mice by age.** All Cebpb^+/-^ mice showed a larger temporal/masseter mass percentage than their Cebpb^+/+^ littermates. The mean of the difference of the temporal/masseter mass percentage between Cebpb^+/-^ and Cebpb^+/+^ amongst young adults (less than 6 months of age), adults (6 to 12 months of age) and senior adults (more than 12 months of age) was 5.66%, 3.29% and 0.55%, respectively. (n = 12, including 6 Cebpb^+/-^ and 6 Cebpb^+/+^ male mice).

The mean weight of the left masseter muscle in Cebpb^+/-^ and Cebpb^+/+^ mice was 113.5 ± 23.9 mg and 120.9 ± 24.4 mg, respectively (p = 0.362) (Table 3). The average weight of the left temporal muscle was 36.6 ± 8.8 mg and 32.0 ± 10.1 mg separately in Cebpb^+/-^ and Cebpb^+/+^ subjects (p = 0.217) (Table 3). The temporal/masseter mass percentage of Cebpb^+/-^ and Cebpb^+/+^ littermates was 32.3 ± 4.9% and 26.1 ± 6.2%, individually (Table 3). The former displayed a larger percentage than the latter (t = 3.841, df = 5, p = 0.012) (Table 3). Figure 5 shows the difference in the temporal/masseter mass percentage between Cebpb^+/-^ and Cebpb^+/+^ mice by age. The mean of the difference of the temporal/masseter mass percentage between Cebpb^+/-^ and Cebpb^+/+^ amongst young adults (less than 6 months of age), adults (6 to 12 months of age) and mature adults (more than 12 months of age) was 5.66%, 3.29% and 0.55%, respectively.

**Table 3:** Means of weight and mass percentage of masseter and temporal muscles by genotypes in paired male littermates

	**Cebpb**^**+/+**^**(n = 6)**	**Cebpb**^**+/-**^**(n = 6)**	**All (n = 12)**	**p value**
Weight of the left masseter muscle (mg)	120.9 ± 24.4	113.5 ± 23.9	117.2 ± 23.3	0.362
Weight of the left temporal muscle (mg)	32.0 ± 10.1	36.6 ± 8.8	34.3 ± 9.4	0.217
Temporal/masseter mass percentage (%)	26.1 ± 6.2	32.3 ± 4.9	29.2 ± 6.2	0.012*

*p < 0.05.

## Discussion

This paper reported phenotypes of mice with Cebpb deficiency, including hypoplastic clavicles, a narrow thoracic cage, a downward tilted zygomatic arch and a comparative mass change of masseter/temporal muscles. In conjunction with our previous findings such as multiple supernumerary teeth and elongated coronoid process in the same species of Cebpb deficient mice [23], these indicated prospective signs of CCD [1-5]. As Cebpb is relevant to expression of Runx2 [18-21] which has been known a determinant of CCD [6], consistency between murine phenotypes of Cebpb deficiency and human manifestations of RUNX2 insufficiency implies a potential effect of Cebpb on occurrence of CCD in mice. Of further note, Hirata *et al* demonstrated a similar osteogenic pattern between Cebpb^-/-^Runx2^+/+^ and Cebpb^+/+^Runx2^+/-^ mice [20]. The former was the same Cebpb^-/-^ genotype used in our study and the latter indicated the genotype of CCD [6]. Thus, the resemblance in impaired osteogenesis indicates an association of Cebpb deficiency with CCD. This shows a potential answer to the CCD cases that were free of RUNX2 mutations as mentioned earlier [6].

The current study found hypoplastic clavicles only in Cebpb^-/-^ mice. This agreed with a recent study showing a similar finding [20]. Furthermore, our study identified a narrower thoracic cage in Cebpb^-/-^ mice and this has never been reported in literature. Since Cebpb expression in clavicles was detected in our sample and ossification of clavicles as well as ribs did not complete until a maturer age [35], delayed bone formation due to Cebpb deficiency [19] could thereby result in clavicular hypoplasia and a narrowed ribcage.

This study has demonstrated for the first time a downward tilt of the zygomatic arch in Cebpb^-/-^ mice. The larger dihedral angle between Z plane and H plane (θ_Z_) identified in Cebpb^-/-^ mice represented that Landmark LZ was located at a more inferior position in the genotype. On the other hand, Landmark LJ and Landmark LS of Cebpb^-/-^ animals were not located at a lower level compared to those of Cebpb^+/+^ and Cebpb^+/-^ mice. This indicated that deformation of the zygomatic arch was limited to the zygoma and not involved with the zygomatic processes of the maxilla and/or the squamosal bone. This finding resembles the feature of a downward tilted zygomatic arch in patients sustaining CCD [3,4]. Although not observed in our mouse model, a past article has suggested an association of human CCD with zygomatic hypoplasia [4]. This may imply a reason why CCD patients displayed a downward inclination of the zygomatic arch. Our detection of Cebpb expression in the zygomatic bone also indicated a potential influence of Cebpb on zygomatic bone formation. Nevertheless, functional interactions among masticatory muscles and craniofacial bones could also contribute to deformity of the zygoma [3,4].

Moreover, this study has reported for the first time a larger temporal/masseter mass percentage in Cebpb^+/-^ mice, which indicated hypotrophy of masseter muscles compared to temporal muscles and/or hypertrophy of temporal muscles compared to masseter muscles. This agreed with a paper which has revealed a volume reduction of masseter muscles in CCD patients [4]. Furuuchi *et al* suggested a causal relationship between hypoplastic zygomatic arch and hypotrophic masseter muscles of CCD cases, based on the anatomic connection [4]. However, this would be difficult to justify the phenotype of masseter muscles in Cebpb^+/-^ mice, since zygomatic deformity was not significant in the genotype. On the other hand, the temporal muscles insert onto the mandibular coronoid process [36] and elongation of the coronoid process in Cebpb^+/-^ mice has been reported by our previous study [23]. Functional activity and muscular development are likely to reciprocally affect growth of the temporal muscle and the coronoid process [3,4]. As morphological and physiological adaptations of temporal muscles after masseter myotomy have been reported [37], hypertrophy of the temporal muscle could also result from compensation for the hypotrophic masseter muscles. Figure 5 illustrating a reduced difference in the temporal/masseter mass percentage between Cebpb^+/-^ and Cebpb^+/+^ mice over age might imply that hypotrophy of masseter muscles and/or hypertrophy of temporal muscles in Cebpb^+/-^ subjects occurred at an early age and the difference was compensated and/or corrected following ageing. Cebpb expression detected in masseter and temporal muscles indicated an association of this gene with both muscles. Of further note, unattainability of Cebpb^-/-^ littermates for assessing masticatory muscles was a limit for our research. Although this was due to a high neonatal mortality of Cebpb^-/-^ mice [24], it compromised the inference of a relationship between abnormality of masticatory muscles and the zygomatic arch. Future investigation in the relationships among Cebpb, bone formation the zygomatic arch and development of masticatory muscles is required.

## Conclusion

This study has reported prospective signs of CCD, including hypoplastic clavicles, a narrowed thoracic cage, a downward tilt of the zygomatic arch and a comparative mass change between masseter as well as temporal muscles, in mice with Cebpb deficiency. The zygomatic deformation was limited to the zygoma and not involved with the zygomatic processes of the maxilla and/or the squamosal bone. In addition, the difference in the temporal/masseter mass percentage between Cebpb deficiency and wild-type mice decreased over age.

Cebpb has been demonstrated as a key regulator for Runx2 which was related to occurrence of most but not all CCD cases. The data presented here, taken together with the authors’ previous study, implicates Cebpb deficiency in some CCD-like phenotypes and this contributes to understanding of the genes involved in the disorder. Succeeding investigation into interactions among Cebpb, Runx2 and musculoskeletal development is indicated.

## Competing interests

The authors declare that they have no competing interests.

## Authors’ contributions

Designing research/study: BH, KT, KB. Performing research/study: BH, PP, HK, YT, KS. Contributing important materials/reagents: KT, MS, SA, AS, KB. Data collection: BH, PP, HK, YT. Data analysis: BH, EJ, PP. Writing paper: BH, EJ. All authors read and approved the final manuscript.
